# Supplementary figures and images for: Shallow epifaunal sea cucumber densities and their relationship with the benthic community in the Okinawa Islands
Source: PeerJ. 2022 Oct 6;10:e14181. doi: 10.7717/peerj.14181 (PMC9548317; doi:10.7717/peerj.14181)

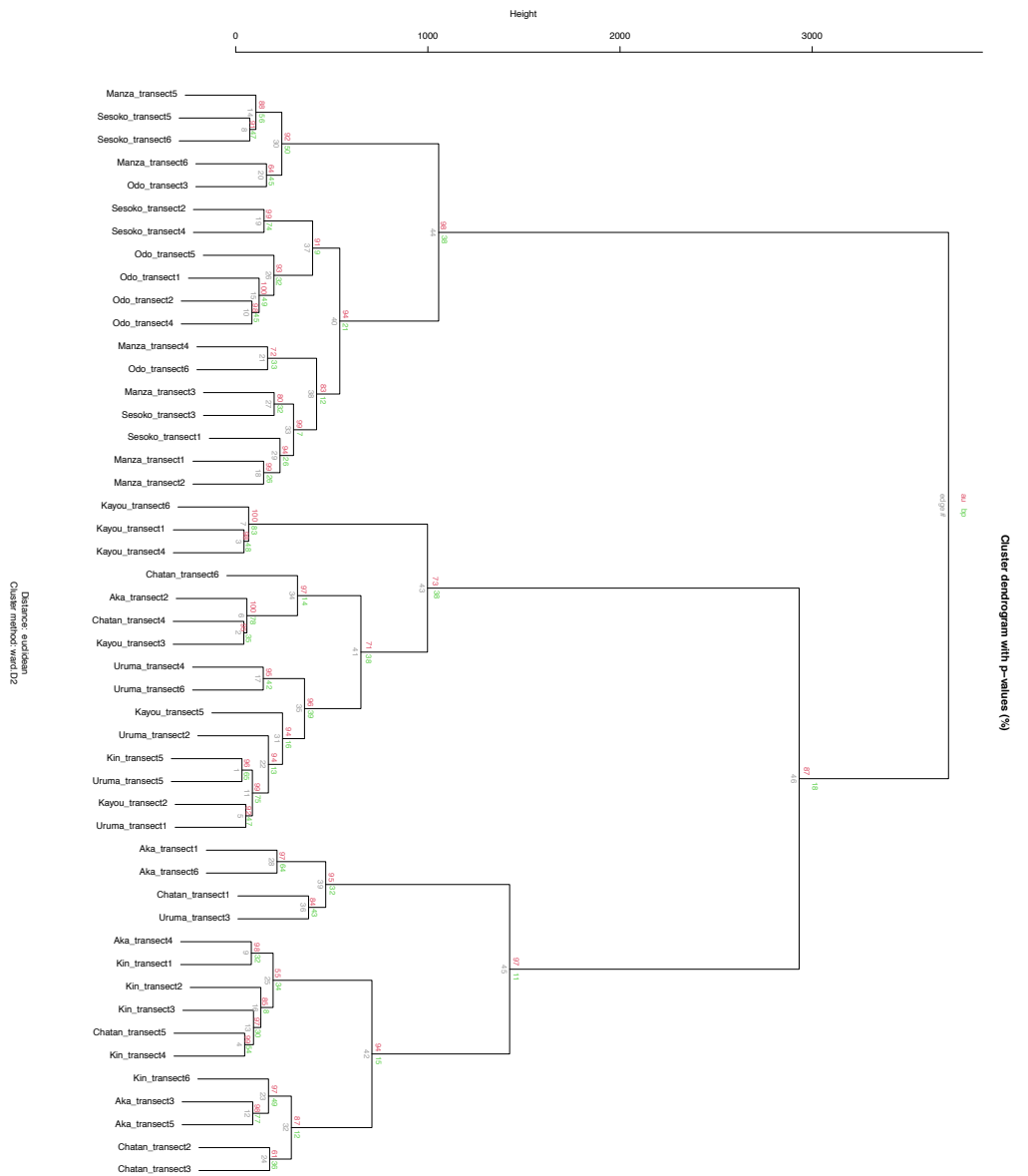

Supplement: Supplemental Information 1 — Numbers at each nodes show p-value (%). [file peerj-10-14181-s001.pdf]

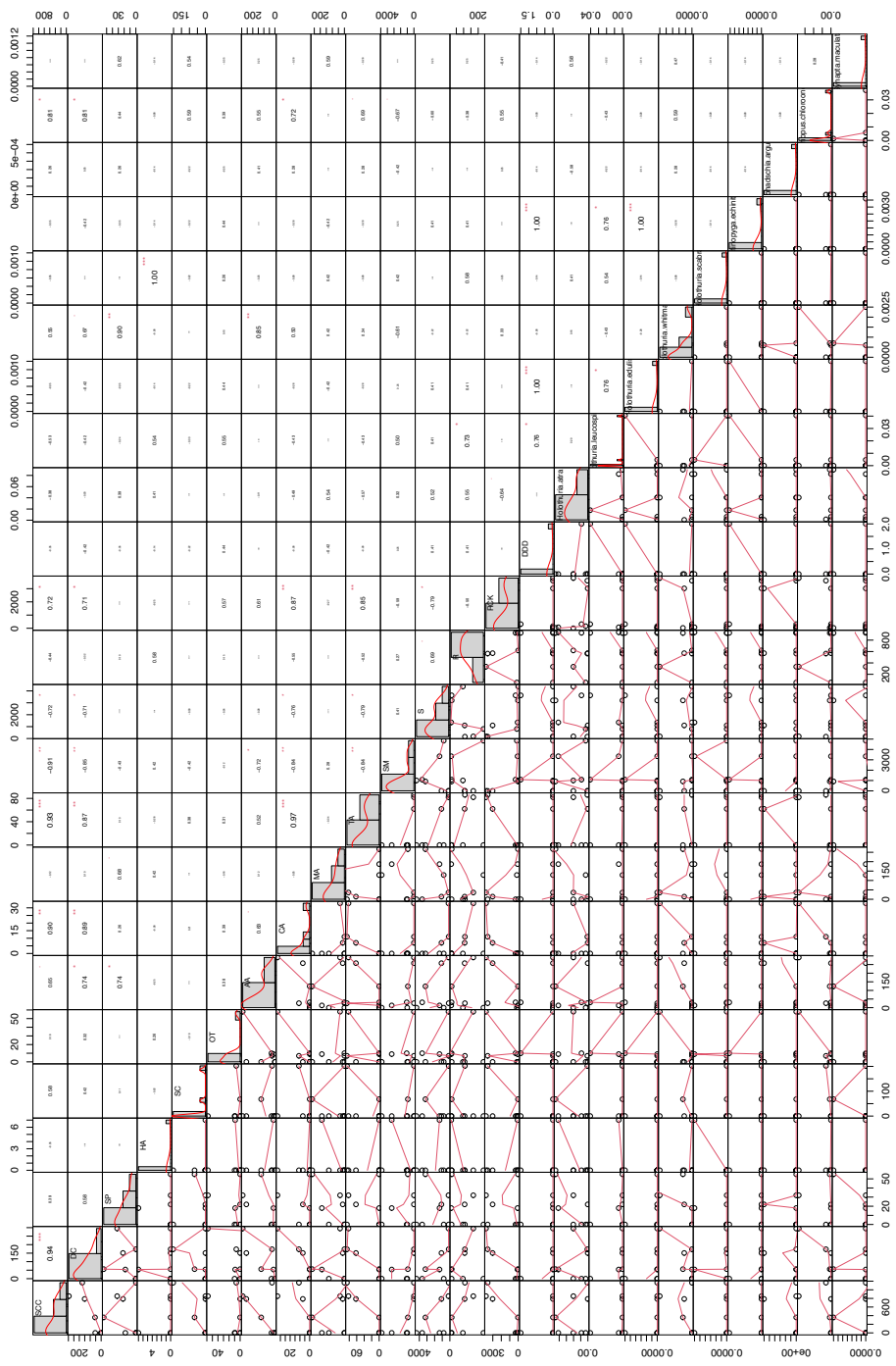

Supplement: Supplemental Information 2 — AA = algal assemblage, CA = coralline algae, DC = dead corals, DDD = unidentifiable components, HA = Halimeda, MA = macroalgae, OT = others, R = rubble, RCK = rocky bottom, S = sandy bottom, SC = soft corals, SCC = scleractinian corals, SM = seagrass meadow, SP = sponges, TA = turf algae. [file peerj-10-14181-s002.pdf]

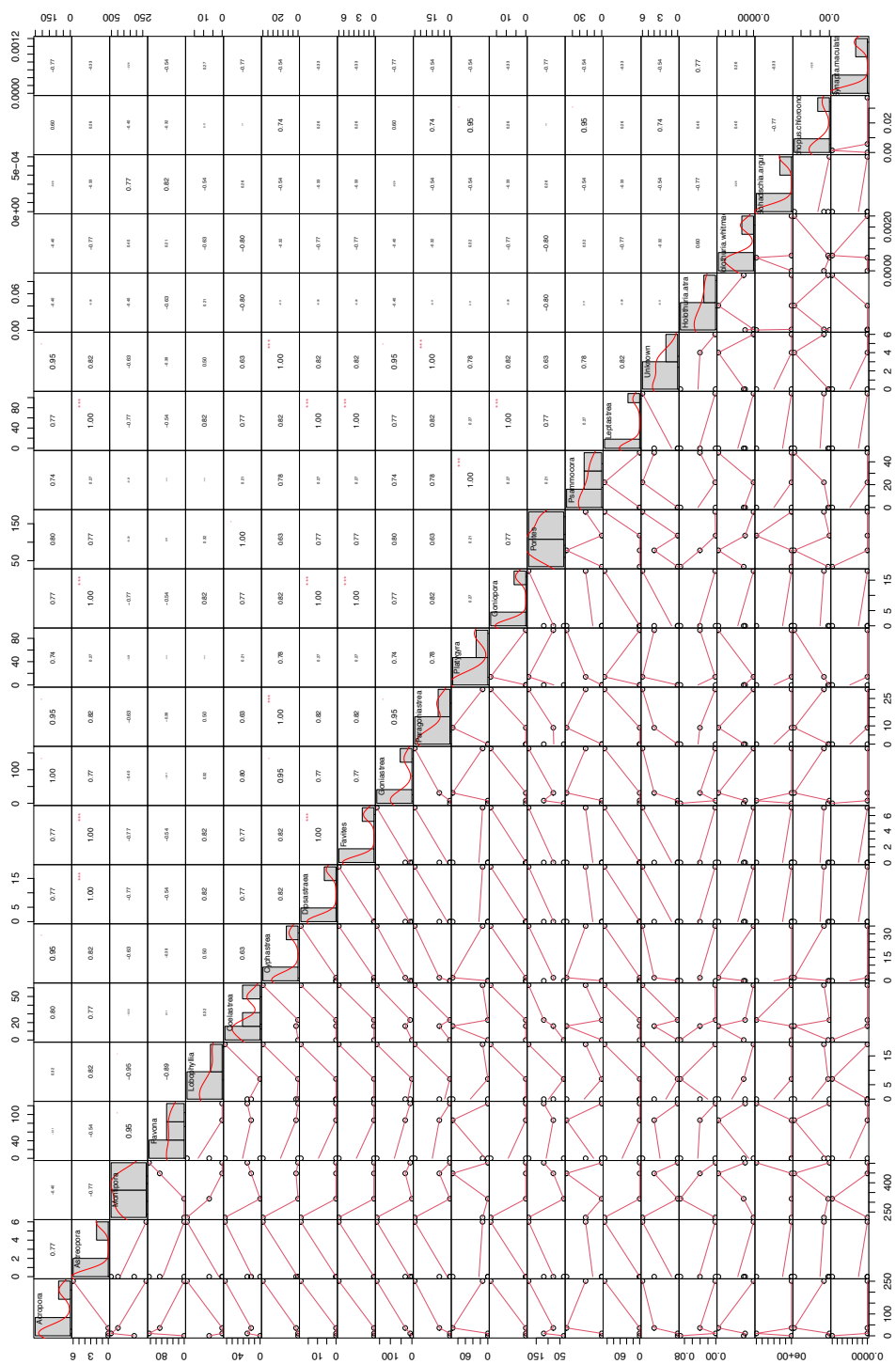

Supplement: Supplemental Information 3 [file peerj-10-14181-s003.pdf]
